# Dextran sulfate sodium and 2,4,6-trinitrobenzene sulfonic acid induce lipid peroxidation by the proliferation of intestinal gram-negative bacteria in mice

**DOI:** 10.1186/1476-9255-7-7

**Published:** 2010-02-01

**Authors:** In-Ah Lee, Eun-Ah Bae, Yang-Jin Hyun, Dong-Hyun Kim

**Affiliations:** 1 Department of Life and Nanopharmaceutical Sciences, Kyung Hee University, 1, Hoegi, Dongdaemun-Ku, Seoul 130-701, Korea

## Abstract

**Abstrect:**

**Background**

To understand whether TLR-4-linked NF-kB activation negatively correlates with lipid peroxidation in colitic animal models, we caused colitis by the treatment with dextran sulfate sodium (DSS) or 2,4,6-trinitrobenzenesulfonic acid (TNBS) to C3H/HeJ (TLR-4-defective) and C3H/HeN (wild type) mice, investigated inflammatory markers, lipid peroxidation, proinflammatory cytokines and TLR-4-linked NF-κB activation, in colon and intestinal bacterial composition in vivo.

**Methods**

Orally administered DSS and intrarectally injected TNBS all caused severe inflammation, manifested by shortened colons in both mice. These agents increased intestinal myeloperoxidase activity and the expression of the proinflammatory cytokines, IL-1β, TNF-α and IL-6, in the colon.

**Results**

DSS and TNBS induced the protein expression of TLR-4 and activated transcription factor NF-κB. However, these colitic agents did not express TLR-4 in C3H/HeJ mice. Of proinflammatory cytokines, IL-1β was most potently expressed in C3H/HeN mice. IL-1β potently induced NF-κB activation in CaCo-2 cells, but did not induce TLR-4 expression. DSS and TNBS increased lipid peroxide (malondialdehyde) and 4-hydroxy-2-nonenal content in the colon, but reduced glutathione content and superoxide dismutase and catalase activities. These colitic inducers increased the number of Enterobacteriaceae grown in DHL agar plates in both mice, although the number of anaerobes and bifidobacteria grown in GAM and BL agar plates was reduced. *E. coli, K. pneumoniae *and *Proteus mirabilis *isolated in DHL agar plates increased lipid peroxidation in liposomes prepared by L-α-phosphatidylcholine, but *B. animalis *and *B. cholerium *isolated from BL agar plates inhibited it.

**Discussion**

These findings suggest that DSS and TNBS may cause colitis by inducing lipid peroxidation and enterobacterial proliferation, which may deteriorate the colitis by regulating proinflammatory cytokines via TLR-4-linked NF-κB activation pathway.

## Background

Inflammatory bowel disease (IBD), including ulcerative colitis and Crohn disease, are chronically relapsing disorders of the intestine [1,2]. Its pathogenic mechanism is assumed to be a dysregulation of the intestinal immune response to intestinal environmental antigens, such as intestinal microflora, and characterized by the activation of lymphocytes, macrophages, enterocytes and endothelial cells, which cause the production of inflammatory mediators, such as IL-1β, IL-6 and TNF-α [3,4]. Intestinal microflora may play an important role in initiating and perpetuating colonic inflammation. Intestinal bacterial endotoxins, such as lipopolysaccharides (LPS), penetrate the epithelial barrier, either due to damage or via paracellular pathways, in order to directly stimulate the mucosal immune system [5]. Alternatively, it is also possible that enteric endotoxins may interact at the apical surface and induce responses in the intestinal epithelial cells. These may result in the production of proinflammatory cytokines, such as TNF-α and IL-1β, and other mediators, such as myeloperoxidase and reactive oxygen species (ROS), causing the inflammatory activation of the mucosal immune system via distinct signaling pathways through Toll-like receptors (TLRs) and/or cytokine receptors [6].

TLRs, which serve as a major link between the innate and adaptive mucosal immune responses, act as transmembrane co-receptors with CD14 in the cellular response to LPS [7]. Among this family of receptors, TLR-4, which is linked in the activation of transcription factor NF-κB, may potentially serve as the main mediators of LPS signaling in the IBD [8]. The activation of NF-κB in mucosal macrophages is accompanied by an increased capacity of these cells to produce and secrete IL-1, IL-6 and TNF-α. These cytokines stimulate NF-kB activation. IBD was not developed or progressed in germ-free IL-10-deficient or IL-2 receptor-deficient animals as well as in TLR-4 knockout animals, by colitic inducers, dextran sulfate sodium (DSS) and 2,4,6-trinitrobenzene sulfonic acid (TNBS) [9-11]. Therefore, the analysis of TLR-linked NF-κB activation, proinflammatory cytokine expression patterns and intestinal gram-negative bacterial quantity in IBD experimental animals could serve as a helpful tool to characterize intestinal inflammation.

Reactive oxygen species (ROS), such as peroxide anion, hydrogen peroxide, and hypochlorous acid, may be involved in the pathogenesis of IBD. The colitic inducers, DSS and TNBS, increase malondialdehyde and 4-hydroxy-2-nonenal (4-HNE) in the colons of both mice, but reduced the glutathione content and superoxide dismutase and catalase activities, as previously reported [12-14]. Treatment with superoxide dismutase significantly ameliorates colitis [15].

In the present study, we conducted experimental colitis by DSS or TNBS in C3H/HeN and C3H/HeJ mice and investigated the relationship between colonic inflammatory markers, such proinflammatory cytokines and lipid peroxidation activation, in colon and colonic gram-negative bacterial quantity.

## Method

### Materials

Dulbecco's modified Eagle medium (DMEM), tetramethyl benzidine, Griess reagent, DSS, hexadecyl trimethyl ammonium bromide, and radio-immunoprecipitation assay (RIPA) lysis buffer were purchased from Sigma Co. (St Louis, MO, USA). A protease inhibitor cocktail was from Roche Applied Science (Mannheim, Germany). ELISA Kits were obtained from Pierce Biotechnology, Inc., (Rockford, IL, USA). Antibodies for of pp65 (phospho-NF-κB), p65 (NF-κB), and β-actin were purchased from Santa Cruz Biotechnology (Santa Cruz, CA, U.S.A.) and Cell signaling technology Inc. (Danvers, MS, U.S.A.), respectively. Enhanced chemiluminescence (ECL) immunoblot system was obtained from Pierce Co. (Rockford, IL, U.S.A.). 4-HNE was purchased from Cayman Chemical Co. (Ann Arbor, MI, U.S.A.).

### Culture of CaCo-2 cells

The human colon cancer cell line Caco-2 (KCLB 30037, Korean Cell Line Bank, Seoul, Korea) was cultured in DMEM supplemented with 10% heat-inactivated FBS, 2 mM L-glutamine, 1% nonessential amino acids, and antibiotics. SW-480 were cultured in Eagle's MEM supplemented with 10% FBS, 2 mM L-glutamine, and antibiotics [16]. Cells were cultured in a water-saturated atmosphere of 95% air and *5% *CO. For immunoblot analysis of NF-κB, TLR-4 and β-actin, 5 × 10^5 ^cells were seeded into a six-well tissue culture plate (2 ml/well) and were incubated at 50 to 70% confluency (normally 1 to 2 days after seeding). The medium was then removed, and the cells were incubated at various times with or without IL-1β. Viability before and after plating was >95% by trypan blue dye exclusion.

### Liposome preparation and lipid peroxidation-inhibitory activity assay

For the liposome preparation, L-α-phosphatidylcholine (0.1 g, type XV-E from egg yolk) was dissolved in diethyl ether (10 ml) and distilled water (0.6 ml) was added. The mixture was sonicated with an ultrasonic disrupter (Eyelar Co., Tokyo, Japan) and evaporated under vacuum on ice. The resulting extract was suspended in 30 ml of 0.1 M N-(2-acetamido)-imidinodiacetic acid (ADA) sodium buffer (pH 6.7), sonicated for 15 min on ice, and centrifuged at 1,500 × g for min at 4°C. The supernatant was used as the liposome suspension.

To assay the lipid peroxidation-inhibitory activity, the liposomal suspension (0.1 ml) was incubated in 1.5 ml of 50 mM sodium phosphate buffer (pH 6.7), 0.1 ml of 2 mM ferrous chloride, and 0.1 ml of 4 mM sodium ascorbate for 2 h at 37°C in the presence or absence of bacterial cells. Lipid peroxide in the reaction mixture was quantified as thiobarbituric acid-reactive substances (TBARS) as reported previously [17].

### Animals

Male C3H/HeN and C3H/HeJ mice (24 - 28 g) were supplied by Orient Experimental Animal Breeding Center (Seoul, Korea). C3H/HeJ mice possess a missense mutation in the TLR-4 gene, which leads to a single amino acid change in the cytoplasmic portion of TLR-4, impeding signal transduction and leading to a phenotype similar to that of TLR-4 knockout mice [18]. C3H/HeJ mice are defective in TLR-4 signaling and in responding to LPS [TLR-4^(LPS-d)^]. All animals were housed in wire cages at 20-22°C and 50 ± 10% humidity, fed standard laboratory chow (Samyang, Seoul, Korea) and allowed water ad libitum. All procedures relating to animals and their care conformed to the international guidelines 'Principles of Laboratory Animals Care' (NIH publication no. 85-23 revised 1985 and Kyung Hee University 2006).

### Preparation of experimental colitis

The colitic mice induced by DSS and TNBS were prepared according to the methods of Dieleman et al. [19] and Neurath et al. [20], respectively.

TNBS-and DSS-induced colitis in C3H/HeN and TLR-4 knockout C3H/HeJ mice was prepared according to the above protocols. These mice were anesthetized with ether and then sacrificed on the 3^rd ^and 7^th ^day after the administration of TNBS and DSS, respectively.

Physical appearance, consistency of feces, diarrhea, the presence of gross blood in stool, and body weight were monitored daily. Macroscopic assessment of the disease grade was scored according to a previously reported scoring system (0, no ulcer and no inflammation; 1, ulceration and local hyperemia; 2, ulceration without hyperemia; 3, ulceration and inflammation at one site only; 4, two or more sites of ulceration and inflammation; 5, ulceration extending more than 2 cm [21], and the colon tissue was then used for immunoblot and enzyme-linked immunosorbent assay (ELISA) analysis.

For histopathological examination, a segment of colon tissues was flash frozen in lipid nitrogen and kept at -80°C for further analysis and another portion was fixed in formalin. Formalin sections were stained with hematoxylin-eosin and evaluated by light microscopy for the presence of lesions.

### Assay of myeloperoxidase activity in colonic mucosa

The colons isolated from the mice were homogenized in a solution containing 0.5% hexadecyl trimethyl ammonium bromide dissolved in 10 mM potassium phosphate buffer (pH 7.0), and then centrifuged for 30 min at 20,000 × g (4°C). An aliquot (50 μl) of the supernatant was added to a reaction mixture consisting of 1.6 mM tetramethyl benzidine and 0.1 mM H_2_O_2_, incubated at 37°C and then the absorbance obtained at 650 nm spectophotometrically time-scanned. The myeloperoxidase activity was defined as the quantity of enzyme degrading 1 μmol/ml of peroxide at 37°C, and expressed in unit/mg protein [22]. The protein content was assayed by Bradford's method [23].

### Assay of lipid peroxide (malondialdehyde), reduced glutathione amount and superoxide dismutase and catalase activities

Lipid peroxidation was estimated in colon homogenates as described by Ohkawa et al. [24]. Briefly, a reaction mixture containing 50 mM Tris-HCl buffer (pH 7.4), 500 μM tert-butyl hydroperoxide (BHP) (in ethanol) and 1 mM ferrous chloride was incubated with the samples at 37°C for 90 min. The reaction was terminated by adding 0.2 ml of 8% sodium dodecyl sulfate followed by 1.5 ml of 20% acetic acid (pH 3.5). The amount of malondialdehyde formed during the incubation was assessed by adding 1.5% thiobarbituric acid and then heating at 95°C for 45 min. After cooling, the samples were centrifuged, and the absorbance of TBARS in the supernatant was measured at 532 nm. The levels of lipid peroxidation are expressed in terms of nmol TBARS/90 min/mg protein.

The amount of reduced glutathione in the tissue homogenate was estimated according to the method of Paglia and Valentine [25]. Catalase and superoxide dismutase activities were estimated according to the method of Prakash et al. [26].

### Analysis of HNE by HPLC

The colon (1 g) was suspended in 1 ml of lysis buffer, homogenized, centrifuged at 13000 rpm for 20 min twice, and the supernatant was analyzed for 4-HNE using HPLC (Younglin high performance liquid chromatography system): column, Develosil ODS-UG-5 (4.6 mm i.d. × 150 mm, 5.8 μm particle diameter); mobile phase, linear-gradient mixture of 10% acetonitrile and 90% acetonitrile for 0 - 20 min and 100% acetonitrile for 20 - 30 min; flow rate, 1 ml/min; and detection, UV at 230/233 nm.

### Enzyme-linked immunosorbent assay (ELISA) and immunoblot

For the ELISA of IL-1β and IL-6, colons were homogenized in 1 ml ice-cold lysis buffer (Radio-immunoprecipitation assay, RIPA) containing 1% a protease inhibitor cocktail and 1% phosphatase inhibitor cocktail). The lysate was centrifuged (15,000 × g, 4 C) for 15 min, and the supernatant transferred to 96-well ELISA plates. IL-1β and IL-6 concentrations were determined using commercial ELISA kits (Pierce Biotechnology, Inc., Rockford, IL, USA).

For the immunoblot of pp65 (phospho-NF-κB), p65 (NF-κB), COX-2, iNOS, TLR-4 and β-actin, the colon tissue was carefully homogenized to obtain many viable single cells, which were resuspended in 1 ml of RIPA lysis buffer containing 1% a protease inhibitor cocktail and 1% phosphatase inhibitor cocktail). After centrifugation, the supernatant was used for the immunoblot assay. The total protein from the collected cells was subjected to electrophoresis on an 8-10% sodium dodecyl sulfate-polyacrylamide gel, and then transferred to a nitrocellulose membrane. The expression levels of pp65 (phospho-NF-κB), p65 (NF-κB), COX-2, iNOS, TLR-4 and β-actin were assayed using their corresponding antibodies, according to a previously reported method [27]. Immunodetection was carried out using an enhanced chemiluminescence detection kit.

### Fecal bacterial suspension preparation and enzyme activity assay

Fresh mouse stools (0.5 g) from each group were collected separately in sterilized plastic cups, carefully suspended in 20-volumes of saline in a cooled tube and centrifuged at 250 × g for 5 min. The supernatant was then centrifuged at 10,000 × g for 20 min. The resulting precipitates were used as the sources for the fecal enzyme assays. All procedures were performed at 4°C.

Bacterial enzyme activity assays for β-glucuronidase and hyaluronic acid degradation were performed according to the method of Lee et al. [28].

### Bacterial culture in mouse stools and their identification

Fresh mouse stools (0.5 g) from each group were collected separately in sterilized plastic cups, carefully suspended in 20-volumes of peptone water, diluted 10-fold in a stepwise manner, and inoculated in agar plates of blood liver medium (BL, Nissui Pharm. Co., Ltd), general anaerobic medium (BL GAM, Nissui Pharm. Co. Ltd) and hydrogen sulfate lactose medium (DHL, Eiken Chem. Co., Ltd). DHL agar plates were cultured anaerobically for 1 day at 37°C, and BL and GAM agar plates were cultured aerobically for 3 days at 37°C.

The colonies grown in DHL agar media were identified by 16S rDNA gene sequencing [29]. Total DNA extracted from the colonies was used as a template to amplify the 16S rRNA gene with primers 27f (5' - AGAGTTTGATCCTGGCTCAG-3') and 1525r (5'-AAAGGAGGTGATCCAGCC-3'), and its sequence was analyzed using BLAST search.

### Bacterial strains and growth conditions

Five intestinal bacterial strains were used in this study: *Bifidobacterium animalis *(B1) and *Bifidobacterium cholerium *(B2) isolated from BL agar plates and *Escherichi coli *(Ec), *Klebsiella pneumoniae *(Kp) and *Proteus mirabilis *(Pm) isolated for DHL agar plates.

Each bacterial strain was grown to an optical density between 3 and 4 at 600 nm in GAM broth, collected by centrifugation (10,000 × g for 30 min) and washed twice with saline. The resulting pellet was used for lipid peroxidation-inhibitory activity assay.

### Statistical analysis

All data are expressed as the mean ± standard deviation, with statistical significance analyzed using one-way ANOVA followed by a Student-Newman-Keuls test.

## Results

**Figure 1:**
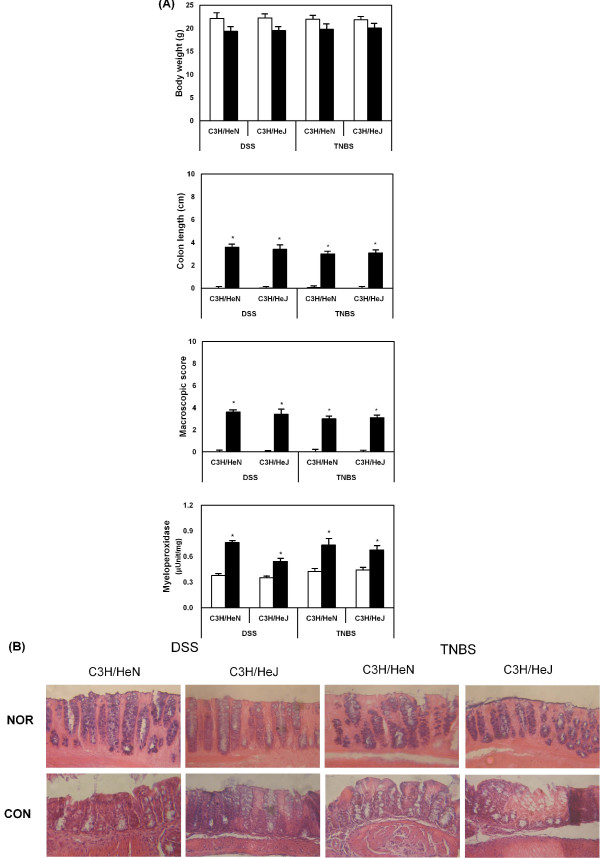
**DSS or TNBS affects body weight (A), colon length (B), macroscopic score (C) and intestinal myeloperoxidase activity (D) in C3H/HeN and C3H/HeJ mice**. A histopathological exam was performed by hematoxylin-eosin staining (E). The mice treated with DSS or TNBS were sacrificed on the 7^th ^and 3^rd ^day, respectively: the white bar, normal group; and black bar, DSS or TNBS-treated group. The values of enzyme activities indicate the mean ± S.D. (n = 7). * Significantly different compared with normal group (p < 0.05).

To evaluate the relationship between TLR-4-linked inflammatory reaction and intestinal gram-negative bacteria, colitis was induced by the oral administration of DSS for 7 days and intrarectal injection of TNBS in C3H/HeN and C3H/HeJ mice, and colitic markers were measured. All normal animals showed body weight gain, but DSS and TNBS treatment reduced body weight gain. These colitic inducers, DSS and TNBS, caused severe inflammation, manifested by shortened, thickened, and erythematous colons in both mice. In macroscopic histology, these inducers showed massive bowel edema and epithelial cell disruption by large ulcerations in both mice (Fig. 1). Myeloperoxidase, an inflammatory marker, was also potently increased in both mice. The colitic inducers also induced lipid peroxide (malondialdehyde) and 4-HNE levels in the colons of C3H/HeN and C3H/HeJ mice, but reduced the glutathione content and superoxide dismutase and catalase activities (Table 1).

**Table 1:** Effect of colitic inducers, TNBS and DSS, on lipid peroxide (malondialdehyde, MDA), 4-hydroxy-2-nonenal (4-HNE), and glutathione (GSH) contents and superoxide dismutase (SOD), and catalase activities in the colons of C3H/HeN and C3H/HeJ mice

	Colitic inducer	Content			Activity	
			
		MDA (μM/mg)	4-HNE(ng/ml)	GSH(μg/ml)	SOD(Unit/mg)	Catalase(mol/min/mg)
C3H/HeN	-^a^	0.74 ± 0.51	0.86 ± 0.85	1.51 ± 1.14	3.76 ± 1.30	5.22 ± 1.33
	DSS	5.60 ± 1.04^#^	11.92 ± 7.01^#^	4.53 ± 0.15^#^	1.06 ± 0.13^#^	0.19 ± 0.94^#^
C3H/HeJ	-^a^	0.72 ± 0.56	2.52 ± 3.11	0.67 ± 0.38	5.21 ± 1.83	6.25 ± 1.21
	DSS	5.39 ± 1.00^#^	2.93 ± 2.26^#^	4.57 ± 0.12^#^	0.72 ± 0.17^#^	0.60 ± 1.41^#^
C3H/HeN	-^a^	0.99 ± 0.09	3.31 ± 0.15	4.92 ± 1.33	3.39 ± 0.34	7.53 ± 0.77
	TNBS	5.11 ± 0.67^#^	15.92 ± 4.03^#^	2.19 ± 0.24^#^	0.16 ± 0.04^#^	1.49 ± 1.00^#^
C3H/HeJ	-^a^	0.66 ± 0.06	1.53 ± 1.80	4.38 ± 0.93	3.64 ± 0.47	4.76 ± 0.65
	TNBS	2.95 ± 0.94^#^	3.57 ± 0.89^#^	2.13 ± 0.25^#^	0.50 ± 0.03^#^	0.82 ± 0.65^#^

The test agents were orally administered once every day for 3 days prior to TNBS treatment. The mice were anesthetized with ether and killed 3 days after TNBS treatment.

^a) ^Normal group treated with vehicle alone instead of colitic inducer.

All values are the mean ± S.D. (n = 10). ^#^Significantly different vs normal group in each column of C3H/HeN or C3H/HeJ mice (P < 0.05).

**Figure 2:**
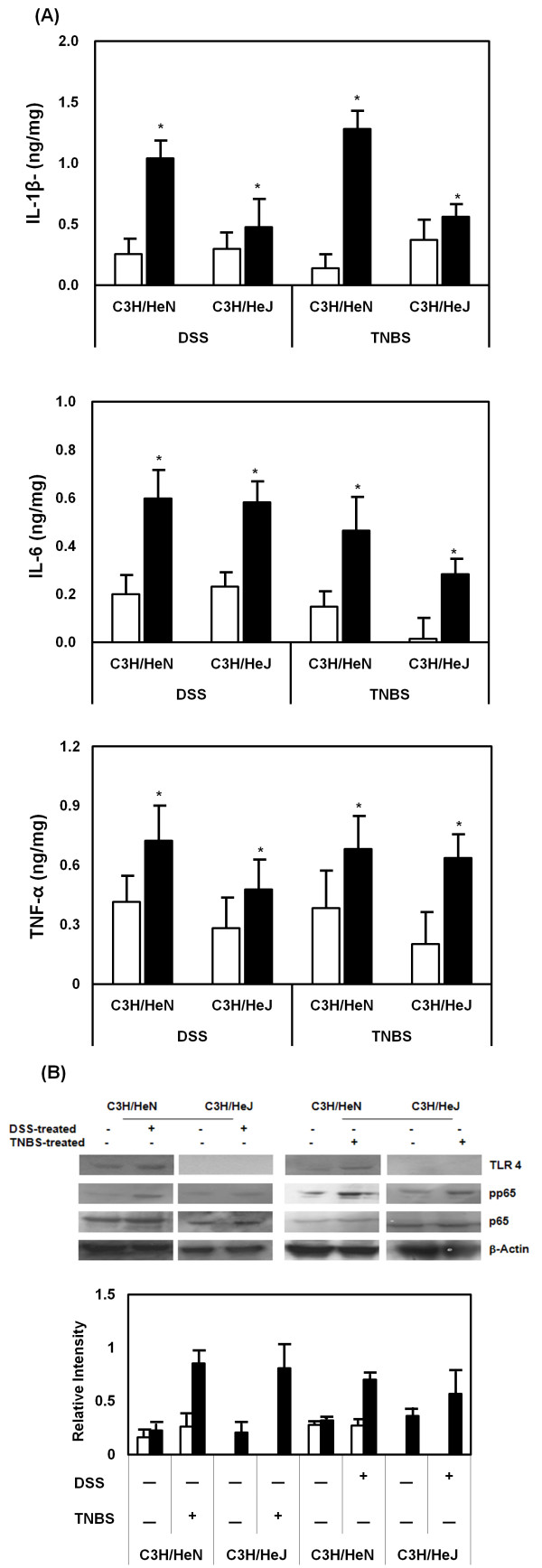
**DSS or TNBS induces proinflammatory cytokine expression and activates transcription factor NF-κB in C3H/HeN and C3H/HeJ mice**. **(A) **Colitic inducers increased the protein expression of IL-1β, IL-6 and TNF-α in the colon of mice. The mice treated with DSS or TNBS were sacrificed on the 7^th ^and 3^rd ^day, respectively: the white bar, normal group; and black bar, DSS or TNBS-treated group. These cytokines were determined by ELISA assays. The values of enzyme activities indicate the mean ± S.D. (n = 7). * Significantly different in each cytokine of DSS or TNBS-treated mice compared with normal group (p < 0.05). **(B) **Colitic inducer increased TLR-4 expression (white bar) and activated NF-κB (black bar). These expressions were measured by immunoblot analysis.

**Figure 3:**
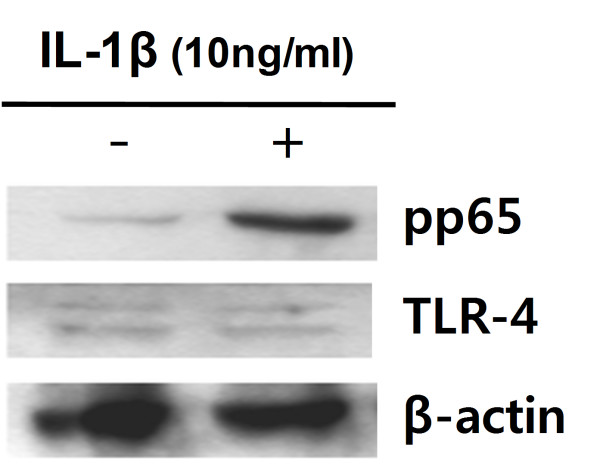
**IL-1β activates transcription factor NF-κB in CaCo-2 cells (5 × 10^5 ^cells)**. The cells were treated with IL-1β (NOR (-), vehicle alone; CON (+), 10 ng/ml IL-1β) for 90 min and then immunoblot for NF-κB (pp65), TLR-4 and β-actin was performed.

Treatment with DSS or TNBS also increased levels of the pro-inflammatory cytokines, IL-6 and TNF-α, in the colon of both mice (Fig. 2). IL-1β was significantly increased in C3H/HeN mice, but not in C3H/HeJ mice. The treatment with these colitic inducers potently induced TLR-4 expression in C3H/HeN mice, and activated NF-κB. However, the treatment with DSS and TNBS in C3H/HeJ did not express TLR-4, although NF-κB was activated. IL-1β potently induced NF-kB in CaCo-2 cells, but did not increase TLR-4 expression (Fig 3).

**Figure 4:**
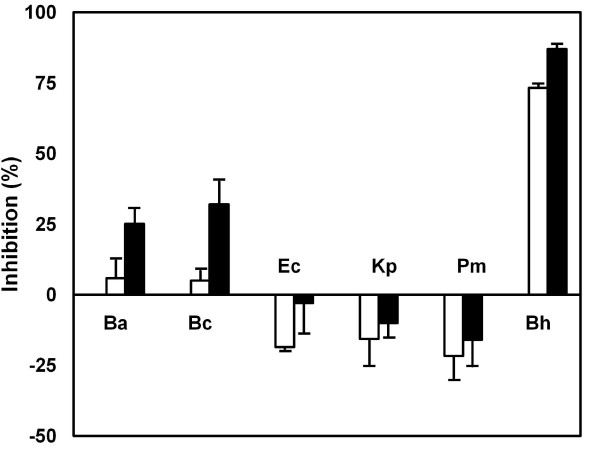
**Intestinal bacteria increase lipid peroxidation in liposomes prepared with L-α-phosphatidylcholine**. Lipid peroxide in liposome was estimated by thiobarbituric acid-reactive substance assay. The intestinal bacteria previously cultured in GAM broth were collected by centrifugation (10,000 × g for 30 min) and washed twice with PBS. The resulting pellet was suspended in phosphate-buffered saline: Ba, *Bifidobacterium animalis*; Bc, *Bifidobacterium cholerium*; Ec, *Escherichia coli*; Lp, *Klebsiella pneumonia*e; Pm, *Proteus mirabilis*. Bh indicates butylated hydroxyl-anisole. Test agents [20 (white bar), and 50 mg/ml (black bar)] was treated. All values are the mean ± S.D. (n = 3).

When C3H/HeN and C3H/HeJ mice were treated with DSS or TNBS, the number of anaerobes grown in GAM agar plate and bifidobacteria in BL agar plate was significantly reduced, but the number of colonies grown in DHL medium, which is a selective medium for Enterobacteriaceae, was significantly increased (Table 2). *Proteus mirabilis, E. coli*, and *K. pneumoniae *were mainly detected in C3H/HeN mice. However, *P. mirabilis *was detected in C3H/HeJ mice, although *E. coli*, and *K. pneumoniae *were detected. Among intestinal bacteria grown in BL and DHL agar plates, bifidobacteria grown, *B. animalis *and *B. cholerium *grown in BL agar plates inhibited lipid peroxidation in liposomes prepared with L-α-phosphatidylcholine, but *E. coli, K pneumonia *and *P. mirabilis *grown in DHL agar plates increased it (Fig. 4).

**Table 2:** Effect of colitic inducers, TNBS and DSS, on number of anaerobes and Enterobacteriaceae in C3H/HeN and C3H/HeJ mice

Mouse	Colitic inducer	Number of colonies grown in agar plate				
		
		GAM (Anaerobes)(×10^10^)	BL (Bifido)(×10^8^)	DHL (×10^6^)		
				
				Ec	Pm	Kp
C3H/HeN	-^a^	3.3 ± 0.5	6.6 ± 0.5	0.9 ± 0.3	0.9 ± 0.3	0.5 ± 0.2
	DSS	2.3 ± 0.7	3.9 ± 0.5^#^	5.1 ± 2.0^#^	2.8 ± 0.6^#^	14.0 ± 2.5^#^
C3H/HeJ	-^a^	1.0 ± 0.4	4.2 ± 0.3	0.9 ± 0.2	-^b^	5.0 ± 0.9
	DSS	1.6 ± 0.5	3.7 ± 1.1^#^	4.7 ± 0.6^#^	-^b^	9.2 ± 0.9^#^

C3H/HeN	-^a^	3.2 ± 0.7	5.9 ± 1.1	0.3 ± 0.04	0.9 0.1	0.7 0.1
	TNBS	1.9 ± 0.8^#^	0.5 ± 0.1^#^	3.2 ± 0.3^#^	1.8 ± 0.1^#^	17.8 ± 2.0^#^
C3H/HeJ	-^a^	1.7 ± 0.6	20.9 ± 2.1	3.5 ± 0.6	-^b^	2.8 ± 0.2
	TNBS	0.6 ± 0.1^#^	7.0 ± 0.8^#^	49.1 ± 4.7^#^	-^b^	31.8 ± 8.6^#^

The test agents were orally administered for 3 days prior to TNBS treatment. The fresh feces was plated in BL, GAM, and DHL agar plates and cultured anaerobically (for BL and GAM agar plates) or aerobically (for DHL agar plates). The colonies grown in DHL agar media were selected and identified by 16S rDNA analysis. Bifido, bifidobacteria; Ec, *Escherichia coli; *Pm, *Proteus mirabilis*; Kp, *Klebsiella pneumonia*.

^a) ^Normal group treated with vehicle alone instead of colitic inducer.

^b) ^Not detected.

All values are the mean ± S.D. (n = 10). ^#^Significantly different vs. normal group in each column of C3H/HeN or C3H/HeJ mice (P < 0.05).

## Discussion

IBD is a severe form of intestinal inflammation, the pathogenesis of which remains to be clearly understood. It is thought that the disease might be attributed to complex mucosal immune responses to resident enteric bacteria[30,31]. The innate immune system recognizes the presence of specific bacterial antigens through pattern recognition receptors [7,8].

TLR-4 is an extensive family of pattern recognition receptors and compelling research has shown that LPS, which is expressed specifically by all gram-negative bacteria, binds to TLR-4 [32,33]. The triggering of TLR-4 complex signaling by LPS results in a cascade of events that leads to the secretion of proinflammatory mediators from monocytes and dendritic cells, which leads ultimately to the activation of an acquired immune response. Signaling through the TLR-4 complex contributes actively to the development of inflammation and may help to maintain an ongoing inflammatory response [34,35]. TLR-4 is potently expressed in intestinal epithelial cells from the colons of patients with IBD [6] and significantly up-regulated during DSS-induced colitis in mice [7].

In the present study, DSS and TNBS caused loss of body weight, and severe inflammation, manifested by shortened, thickened and erythematous colons in both C3H/HeN and C3H/HeJ mice. DSS and TNBS did not only potently activate NF-κB, but also induced the expression of TLR-4 and proinflammatory cytokines IL-1β, TNF-α and IL-6 in C3H/HeN mice, as previously reported [36,37]. However, DSS and TNBS-induced TNF-α and IL-1β expression less in C3H/HeJ mice than C3H/HeN mice, although NF-κB activation was potently activated in both mice. DSS and TNBS caused more severe colonic inflammation and colon shortening in C3H/HeN mice than C3H/HeJ mice. Treatment with IL-1β in CaCo-2 cells potently activated NF-κB, but did not induce TLR-4 expression. These results suggest that DSS and TNBS may induce colitis via the expression of IL-1β, IL-6, and TNF-α, and that IL-1β may be dependent on TLR-4-linked NF-kB activation.

Intestinal bacterial endotoxins, such as gram-negative lipopolysaccharides, activate TLR-4-linked NF-kB and cause colitic inflammation [7,16,30]. To confirm the role of intestinal bacteria in colitic inflammation, we measured colonic bacterial composition in DSS or TNBS-treated mice. When C3H/HeN and C3H/HeJ mice were treated with DSS or TNBS, the number of anaerobes and bifidobacteria was significantly reduced, but the number of colonies grown in DHL medium, which is a selective medium for Enterobacteriaceae, was significantly increased. *Proteus mirabilis, E. coli *and *K. pneumoniae *were mainly detected in C3H/HeN mice, and *E. coli *and *K. pneumoniae *were detected in C3H/HeJ mice. These gram-negative bacteria, *Proteus mirabilis, E. coli *and *K. pneumoniae*, produces endotoxin, which activates TLR-4-linked NF-kB. Treatment with DSS or TNBS increased the number of gram-negative bacteria and decreased the number of bifidobacteria and anaerobes. DSS and TNBS caused more severe colitis in C3H/HeN mice than in C3H/HeJ mice, which paralleled changes in bacterial composition. Thus, treatment with DSS and TNBS may increase the growth of gram-negative bacteria, produce LPS, and cause colitis via TLR-4-linked NF-kB activation. However, DSS and TNBS caused colitis in C3H/HeJ mice, which is TLR-4-defective, as well as in C3H/HeN mice. These results suggest that DSS and TNBS may cause colitis via TLR-4-linked pathway as well as other pathway(s), such as oxidative stresses. Reactive oxygen species (ROS), such as peroxide anion, hydrogen peroxide, and hypochlorous acid, may actually be involved in the pathogenesis of IBD as treatment with superoxide dismutase significantly ameliorates the colitis [15]. DSS and TNBS increased malondialdehyde and 4-HNE in the colons of both mice, but reduced the glutathione content and superoxide dismutase and catalase activities, as previously reported [12-14]. Particularly, these colitic inducers dramatically increased 4-HNE levels in C3H/HeN, compared with those in C3H/HeJ. These results suggest that DSS and TNBS may cause colitis independently of TLR-4-linked NF-κB activation, and lipid peroxidation in colitis may be induced by TLR-4-linked NF-κB activation.

Enterobacteriaceae, *E. coli, K. pneumoniae *and *P. mirabilis *induced by the treatment with DSS or TNBS in mice increased lipid peroxidation in liposomes prepared by L-α-phosphatidylcholine, but *Bifidobacteria *inhibited it. The results suggest that the colitic inducers, DSS and TNBS, may induce ROS directly as well as indirectly via the proliferation of Enterobacteriaceae. Based on these findings, DSS or TNBS may cause colitis by lipid peroxidation and enterobacterial proliferation, which may deteriorate the colitis by regulating proinflammatory cytokines via TLR-4-linked NF-κB activation pathway.

## Abbreviations

(DSS): Dextran sulfate sodium; (TNBS): 2,4,6-trinitrobenzenesulfonic acid; (TLR4): Toll-like receptor 4; (LPS): Lipopolysaccharides; (4-HNE): 4-hydroxy-2-nonenal; (TNF alpha): Tumor necrosis factor alpha; (IL-6): Interleukin-6; (ROS): Reactive oxygen species.

## Competing interests

The authors declare that they have no competing interests.

## Authors' contributions

IA performed all of the experiments. EA performed the immunoassay into cell line. YJ performed the identification of intestinal bacteria. DH conceived of the study, and performed its design and coordination. All authors have read and approved the final manuscript.
